# Wound Complications after 2-Octyl Skin Closure Systems for Total Joint Arthroplasty

**DOI:** 10.7150/jbji.42079

**Published:** 2020-04-06

**Authors:** Andrew Michalowitz, Robert Comrie, Christopher Nicholas, Michael Wagner, James Kehoe

**Affiliations:** McLaren Macomb Hospital

**Keywords:** 2-cyanoacrylate, 2-octyl, Dermabond, Prineo, Exofin, total joint arthroplasty, dermatitis, superficial infection

## Abstract

**Introduction**: Total joint arthroplasty is projected to expand rapidly by 2030. With large numbers of patients undergoing TJA, the choice of incisional closure has come into question. We compared the 2-Ocyl cyanoacrylate closure system of Dermabond ® Prineo ® with Exofin Fusion ® to compare rates of adverse wound outcomes after total joint arthroplasty. Secondary outcome measures were age, sex, and medical comorbidities between groups.

**Methods**: We retrospectively reviewed adverse wound outcomes with skin closure in TJA in 281 patients (160 Dermabond Prineo and 121 Exofin Fusion). Clinical charts were analyzed out to the 6-week post-op visit.

**Results**: The rate of overall adverse superficial wound outcomes was similar between the two groups with Dermabond Prineo (N=20) and Exofin Fusion (N=19). The rate of cellulitis was significantly higher for Dermabond Prineo when compared to Exofin Fusion (P=0.033). No other significant differences were found for rate of superficial or deep wound complications or for secondary outcomes.

**Conclusions**: The two 2-octyl wound closure systems had similar adverse superficial wound complications. Except for Dermabond Prineo having a higher rate of post-operative cellulitis, there were no statistically significant differences for other superficial or deep adverse wound outcomes or secondary outcomes. A future randomized control trial or prospective cohort study is needed for a more robust analysis.

## Introduction

The demand for total knee and hip arthroplasty has exploded in the United States and is projected to grow 174% for total hip arthroplasty (THA) and 673% for total knee arthroplasty (TKA) to total almost four million annual combined procedures by 2030^1^. Periprosthetic joint infection (PJI) is reported between 2% and 0.4% for patients undergoing TKA in a Medicare population of 69,663 patients within two years and between two to 10 years, respectively^2^. Literature on risk of deep infection with presence of superficial wound infection after total joint arthroplasty is mixed. Carroll, et al. show that signs of superficial wound infections such as surgical site infections and prolonged drainage may lead to a 35 times increase risk of deep PJI, while Guirro, et al. report that of 45 superficial wound infections in 3000 primary TKAs treated with oral antibiotics with or without surgical debridement, none led to deep PJIs at 70 months of followup^3^,^4^. The choice of incisional closure is also a controversial area.

There are many choices of skin incision closure systems for total joint arthroplasty (TJA). These include staples, suture, or newer methods of absorbable barbed suture with a tape and glue overlay to seal the incision for the first 10 to 14 postoperative days. Dermabond ® tissue adhesive is a liquid 2-octyl-2-cyanoacrylate adhesive (Ethicon, Inc., Somerville, NJ, USA) which is combined with Prineo ® tape (Ethicon, Inc., Somerville, NJ, USA), a pressure-sensitive, self-adhering polyester mesh FDA approved for skin closure in TJA^5^. The Exofin ® Fusion wound closure system combines a similar 2-octyl-2-cyanoacrylate liquid monomer adhesive with a non-woven polyester mesh. The Exofin Fusion system has a higher viscosity adhesive than that of Dermabond at 460 and eight centipoise, respectively^6.^ Both systems have proprietary chemical additives but do include D & C Violet Dye #2 which can cause allergic dermatitis. According to “data on file,” the newer Exofin Fusion system from Chemence Medical, Inc. has a drying time of 38 seconds compared to 60 seconds for the Dermabond/Prineo tape system^6^. Both types of wound closure systems were combined with a 3-0 Stratafix ® monocryl (Ethicon, Inc., Somerville, NJ, USA) barbed suture in the subcutaneous tissue in this study^5^. No previous study to date has compared the adverse wound outcomes of the Exofin Fusion wound closure system to the extensively studied 2-octyl-2-cyanoacrylate Dermabond glue and Prineo tape system.

The purpose of this retrospective chart review was to search for adverse skin reactions and surgical site infections in patients receiving 2-octyl-2-cyanoacrylate glue and tape with either Dermabond glue and Prineo tape or Exofin Fusion glue and tape system after total knee and total hip arthroplasty. The primary outcome was to find and record adverse events related to wound complications with these skin incision closure systems. They are subdivided into superficial (suture abscess, skin breakdown, cellulitis, allergic dermatitis, need for oral antibiotics, and/or persistent drainage) and deep (capsule dehiscence, need for IV antibiotics, and any need for irrigation/debridement or revision surgery) complications. Secondary outcome measures were patient data related to these events including age and sex, as well as for medical comorbidities including smoking status, BMI, diabetes mellitus, and presence of liver or kidney disease.

## Methods

Electronic health records from April 1, 2017 to April 1, 2019 were searched in the McLaren McKesson system. Any patient receiving a primary total hip or knee arthroplasty during this date range with the orthopedic surgery attendings listed as investigators at McLaren Macomb Hospital were included in the study. Exclusion criteria included revision arthroplasty, active infection in area of incision prior to primary surgery, malignancy within surgical field, prior hip or knee surgery, arthroplasty performed for acute fracture, staple closure, follow-up of less than six weeks, or history of allergic skin reaction to adhesives or tape. Follow-up data was retrieved from the office charts of the attending surgeon investigators, and follow-up was observed until six weeks post-operatively as this was the average follow-up time.

All THAs were performed through an anterior approach on a Hana table or anterolateral approach on a standard table. All TKAs were performed through a standard median parapatellar approach. In all patients, the subcutaneous layer was closed with simple interrupted 2-0 vicryl and the skin was closed with a knotless, barbed STRATAFIX^TM^ monocryl. One of the two 2-octyl closure systems was then placed superficially. From April 1 2017 to April 1 2018, our institution used the Dermabond Prineo system for TJA closures. All patients after this point received an incisional closure with the Exofin Fusion system due to cost optimization. Surgeon preference was not involved in this decision. A staple control group was not included since there was only one surgeon at our institution utilizing staples as a primary skin closure method and it was felt that his inclusion could lead to confounding.

Statistical analysis was performed with STATA (StataCorp. 2015. *Stata Statistical Software: Release 14*. College Station, TX: StataCorp LP). Due to the infrequency of the superficial and deep wound complications, the Fisher Exact test was used for ordinal and binary variables by comparing frequency and student's t test was used for larger sample sizes. Descriptive statistics were generated for continuous data recorded for secondary measures.

As this is a pilot study with only 281 cases performed within the above time frame, logistic regressions were not appropriate. Subgroup analyses were performed to determine the effect of total wound complications in relation to secondary outcomes (age, sex, comorbidities). No power analysis was performed prior to the start of the study but a similar study by Campbell, et al determined that 189 patients per study group was required to be appropriately powered^6^.

## Results

323 primary total hip and knee arthroplasties were performed by the study authors during the above time period. A total of 29 patients were excluded due to closure with skin staples, three patients due to fracture as their reason for arthroplasty, six patients for follow-up less than six weeks, and four patients due to wound closure with Prineo glue without tape. With 42 patients excluded, a total of 281 patients were included for evaluation which comprised a combination of total hip and total knee arthroplasties from the above dates. There were 160 patients in the Dermabond/Prineo control group and 121 patients in the Exofin Fusion experimental group.

### Primary Outcomes: Superficial and Deep Wound complications

The rate of overall superficial wound complications was similar for the two groups with Dermabond/Prineo (N=20) and the Exofin Fusion system (N=19). Between the subgroups of superficial wound complications, the number of cases of cellulitis was significantly higher for Dermabond/Prineo tape when compared to the Exofin Fusion system (P = 0.033). No significant differences were found between the two for aggregated superficial wound complications, skin breakdown, suture abscess, allergic dermatitis, persistent drainage, need for oral antibiotics, or office debridement. There were also no significant differences found for total deep wound complications or for the subgroups of capsule dehiscence, need for IV antibiotics, draining sinus tract, or need for surgical debridement or revision (Table 1).

### Secondary Outcomes: Patient data and co-morbidities

The mean age of patients in the two wound closure system groups was nearly identical with 65.6 and 65.7 for Prineo and Exofin, respectively. No statistical significance was found between age, sex, or BMI and rate of any superficial or deep wound complication. There was also no statistical significance found for history of diabetes mellitus, active smoking, or chronic renal or liver disease. The data is listed in (Table 2).

## Discussion

With the anticipated dramatic increase in expected total joint arthroplasties in the next decade, the rate of superficial and deep wound complications is also expected to increase proportionately. Most studies looking at these complications focused on staples compared to suture or one of the newer tape and glue systems. Many analyzed wound complications for knotless barbed suture compared to standard absorbable suture for skin closure with mixed results in prospective randomized control trials despite a universal reduction in closure time^7^-^14^. In a prospective randomized control trial by Khan, et al., the Dermabond skin closure group showed less drainage within the first 24 hours but more overall in follow-up when compared to monocryl suture^15^.

Rash and allergic dermatitis are not infrequent complications with the use of Dermabond with and without Prineo tape^16^-^18^. When applied to the skin incision after closure with a subcutaneous suture, 2-cyanoacylate monomers polymerize into long chains to form an antimicrobial barrier in the presence of anionic substances such as blood and moist skin^19^. This barrier forms a protected moist environment which has been shown to be ideal for surgical wound healing. 2-cyanoacrylate adhesives degrade to formaldehyde and cyanoacetate after application. Although formaldehyde is also a skin irritant, most breakdown occurs after the adhesive has sloughed off the skin^19^.

The finding of a statistically and clinically significant difference between Prineo tape (N=4) and Exofin (N=0) in the number of cases of cellulitis is surprising. However, we also found almost double the number of patients in the dermabond group had diabetes as a comorbidity as compared to the Exofin group (N=33 vs N= 17). Diabetes has been shown to limit wound healing and lead to superficial and deep wound complications. The finding could also be explained by a weakening of the interface between the 2-octyl glue and tape of the Prineo system as compared to the Exofin Fusion system which may have led to increased drainage. This could theoretically cause an increased incidence of cellulitis. However, due to the proprietary nature of these two products, it is difficult to ascertain more information about their physical properties and interactions.

The comorbidities studied for secondary outcomes were chosen since they have been shown to lead to an increased risk of perioperative joint infection. BMI greater than 40, chronic renal/liver disease, and smoking status all have been shown to lead to poor wound healing after TJA^20^,^21^.

There are over 51 randomized control trials and more than 300 preclinical and clinical studies looking at the safety and effectiveness of Dermabond tissue adhesive^7^. To date, there are no studies on the effectiveness or adverse wound complications of the Exofin Fusion system. A strength of this study is that all attending surgeons and residents had utilized the Dermabond glue/Prineo tape system for 18 months until April 2018 at which point the hospital replaced it with the Exofin Fusion system. Therefore, the learning curve for the use of each system was similar and there was no selection bias for the use of the two systems. In addition, all patients received the same type of subcutaneous and skin closure and all capsule and skin closures were performed by majority senior residents in both groups.

Limitations to this study are those that are inherent in retrospective cohort studies. The data was limited by the descriptive nature of the office notes of the attending surgeons involved in the study. A patient was recorded as having cellulitis or one of the other wound complications only if expressly written in the chart**.** Terse charting or the possible reluctance to comment on questionable wound healing in office notes limited our interpretation. Prospective, objective guidelines for each wound complication variable is planned for a future study and would provide a more robust data set for analysis. Also, a comparative prospective study of the above wound closure systems with either staples or suture alone could also be conducted in the future. Since insufficient time has passed since the Exofin system replaced the Dermabond Prineo system to accrue enough cases at our institution to satisfy the power calculation in either group (N=189), a future study with a larger sample size would be of benefit. Also, paper office charts were difficult to locate past 2 years at our institution making it difficult to analyze Prineo tape patients from this period. Interestingly, a study by Almustafa, et al retrospectively reviewed a cohort of 3,932 TKAs and risk of surgical site infection with various closure systems. They found that there were not enough cases of infection in the subgroups to run logistic regressions despite their large cohort^13^.

Finally, the low number of wound complications inherent to these wound closure systems makes their study difficult without large patient groups. This was why total knee and hip arthroplasty were not subdivided between superficial and deep wound complications for analysis. Despite this limitation, the authors still felt that a statistical analysis was appropriate for reference.

## Conclusion

The study of wound closure is an evolving area in orthopedics. An ideal wound closure system after total joint arthroplasty would be low-cost, expediently applied, and minimize both superficial and deep wound complications. This is the first study to date to look at wound complications after TJA with the Exofin Fusion system. The results of this study show that the Dermabond/Prineo and Exofin Fusion systems both show a similar amount of superficial and deep wound complications and are safe for use in total joint arthroplasty. However, a future investigation via randomized control trial or prospective cohort study would lead to a more robust analysis of this important area of wound healing.

## Figures and Tables

**Table 1:** Primary Outcomes: Superficial and Deep Wound Complications

Skin Closure System	Dermabond/Prineo (N=160)	Exofin Fusion (N=121)	P
Superficial Wound Complication	19.2% (N=20)	18.0% (N=19)	0.489
Deep Wound Complication	2.5% (N=3)	0.6% (N=1)	0.318
Cellulitis	3.3% (N=4)	0 (N=0)	0.033
Skin Breakdown	5.1% (N=6)	1.9% (N=3)	0.18
Suture Abscess	5.9% (N=7)	3.1% (N=5)	0.374
Allergic Dermatitis	1.6% (N=2)	1.9 (N=3)	1.00
Persistent Drainage	6.9% (N=8)	5.8% (N=9)	0.803
Capsule Dehiscence	0.8% (N=1)	0 (N=0)	0.431
Draining Sinus Tract	0.8% (N=1)	0 (N=0)	0.431
IV Antibiotics	1.6% (N=2)	0 (N=0)	0.185
Oral Antibiotics	5.1% (N=6)	3.1% (N=5)	0.539
Need for Revision Surgery	1.6% (N=2)	0.6% (N=1)	0.579
Office Debridement	2.5% (N=3)	1.2% (N=2)	0.655

**Table 2:** Secondary Outcomes: Patient Data and Co-Morbidities

Variable	Dermabond/Prineo (N=160)	Exofin Fusion (N=121)	P
**Age (Years)**	65.6 (42-83)	65.7 (42-91)	0.888
**Gender**			0.86
Male	66	59	-
Female	94	62	-
**Body Mass Index**	32.1	30.8	0.771
**Smoking**	18	19	0.447
**Diabetes**	33	17	1.0
**Chronic Renal Disease**	6	10	0.06
**Liver Disease**	4	1	0.539
